# An exo-cell assay for examining real-time γ-secretase activity and inhibition

**DOI:** 10.1186/1750-1326-4-22

**Published:** 2009-06-02

**Authors:** Christopher C Shelton, Yuan Tian, Mark G Frattini, Yue-Ming Li

**Affiliations:** 1 Molecular Pharmacology and Chemistry Program, Memorial Sloan-Kettering Cancer Center, New York, NY 10065, USA; 2 Department of Medicine, Memorial Sloan-Kettering Cancer Center, New York, NY 10065, USA; 3 Department of Pharmacology, Weill Graduate School of Medical Sciences of Cornell University, New York, NY 10021, USA; 4 Department of Physiology, Biophysics and Systems Biology, Weill Graduate School of Medical Sciences of Cornell University, New York, NY 10021, USA

## Abstract

γ-Secretase is an aspartyl protease that cleaves multiple substrates that are involved in broad biological processes ranging from stem cell development to neurodegeneration. The investigation of γ-secretase has been limited by currently available assays that require genetic or biochemical manipulation in the form of substrate transfection or membrane preparation. Here we report an exo-cell assay that is capable of characterizing γ-secretase activity in any cellular system without limitation. Using a highly active, recombinant substrate this assay can quickly and easily ascertain the status of γ-secretase activity in cell systems and patient samples. We have applied this method to determine the activity of γ-secretase in primary cell samples where transfection and/or membrane isolation are not viable options. Importantly, it allows for the detection of real time γ-secretase activity after inhibitor or drug treatment. The application of this assay to determine the role of γ-secretase in physiological and pathological conditions will greatly facilitate our characterization of this complex protease and help in the development and evaluation of γ-secretase-targeted therapies in Alzheimer's disease or a variety of neoplasms.

## Background

γ-Secretase is a multi-subunit protease that executes an extraordinary cleavage of substrates within the lipid bilayer. This process of target hydrolysis within the membrane environment is known as regulated intramembrane proteolysis (RIP) [1] whereby cleavage by γ-secretase releases a protein fragment from its membrane tether that can then transmit its signal. γ-Secretase was originally identified as the enzyme responsible for cleavage of the amyloid precursor protein (APP) [2]. Cleavage of APP generates β-amyloid peptides that are believed to play a causative role in the neuropathogenesis of Alzheimer's disease [3] according to the "amyloid cascade hypothesis." Additionally, it has been determined that γ-secretase cleaves a multitude of other substrates that include the Notch receptors [4], ErbB-4 [5], CD44 [6], as well as the Notch ligands Delta-1 and Jagged-2 [7,8] amongst others. Deregulated Notch signaling has been associated with the development of various cancers, including T-cell Acute Lymphoblastic Leukemia (T-ALL) [9]. Due to the central role of γ-secretase in these pathologies, considerable efforts have been made to characterize this unique protease.

In order to better understand γ-secretase, *in vitro *assays using purified exogenous recombinant substrate [10] or assays utilizing isolated membrane from systems overexpressing substrate have been developed and reported [11,12]. Currently, there are two predominant options to study this protease in a cell line of interest: 1) stably transfect the cell line with plasmids encoding APP, Notch or other substrate fragments and conduct whole-cell based detection assays, or 2) obtain large quantities of the cell line and isolate the membrane fraction in a time-consuming process. This can then be examined using an *in vitro *assay that employs exogenous recombinant substrate as mentioned previously. Due to these limitations, it is often an extremely challenging task to quickly characterize γ-secretase activity in multiple cell lines and primary cells. Furthermore, it is currently impossible to examine the real-time effect of various treatments on the status of γ-secretase in cell systems without stable transfection. For instance, treatment of a Notch-dependent cell line with γ-secretase inhibitors may have an anti-proliferative effect, but available methods cannot ascertain the extent of real-time γ-secretase inhibition in the system. Therefore, development of an assay that does not require transfection or membrane preparation and is applicable for any cell type has become an urgent issue for defining the relationship of γ-secretase inhibition and its biological responses. This is particularly critical to evaluate γ-secretase inhibitors being used in preclinical and clinical studies because assessment of target inhibition will facilitate the identification and establishment of effective therapies. Recently, we have determined that the use of biotinylated substrate greatly enhanced substrate activity and assay sensitivity over previous versions [13]. This prompted us to apply a similar strategy to the development of a simplified γ-secretase assay capable of quantifying real-time activity in cell-based systems.

In this study we have developed a novel γ-secretase assay that does not require membrane preparation and/or substrate plasmid transfection. This γ-secretase assay that we refer to as an "exo-cell" assay applies a highly active, biotinylated recombinant substrate (Sb4) of γ-secretase exogenously to cells in the presence of a small quantity of CHAPSO detergent. We have found that this 96-well assay format can detect γ-secretase activity from as little as a few thousand cells. Furthermore, we can easily detect γ-secretase activity from primary B-cell Chronic Lymphocytic Leukemia (B-CLL) cells isolated from patients. More importantly, this assay can monitor the real-time γ-secretase activity in a 96-well format after inhibitor treatment and has allowed us to establish a correlation between the anti-proliferative effect of γ-secretase inhibitors against lymphoma cells and real-time reduction in γ-secretase activity. Taken together, the development of this novel assay allows for the characterization of real-time γ-secretase activity directly in cell lines as well as primary patient samples. This assay will simplify the study of γ-secretase and provide new tools in the characterization of this enzyme as well as facilitate the development of therapies against Alzheimer's disease and Notch-dependent neoplasms. Furthermore, the application of this simplified method will greatly enhance our ability to examine this unique enzyme and advance our understanding of γ-secretase biology.

## Results

### Development of an exo-cell γ-secretase assay using a biotinylated recombinant APP substrate

We recently demonstrated that it was possible to directly biotinylate a γ-secretase peptide substrate to be utilized in an *in vitro *assay [13]. Here, we have designed a truncated, recombinant APP protein that is directly biotinylated during overproduction in *Eschericia coli*. This substrate is highly active and offers an advantage to develop an easy and sensitive γ-secretase assay. This allows for the elimination of stable transfection of γ-secretase substrate into the cell line of interest or isolation of membrane from large numbers of cells that can then be examined using an *in vitro *γ-secretase assay.

**Figure 1:**
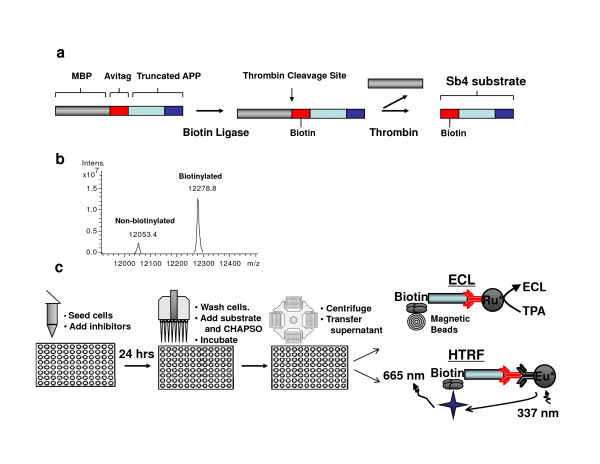
**Recombinant γ-secretase substrate allows for detection of protease activity directly in cells**. (a) Sb4 γ-secretase substrate. Schematic of the truncated Sb4 substrate from the amyloid precursor protein that has an engineered MBP tag as well as Avitag for purification and biotinylation, respectively. A thrombin cleavage site between the MBP tag and avitag allows for the removal of MBP by thrombin treatment following substrate purification. (b) LC-MS analysis identified Sb4 at the expected size and determined that greater than 90% of purified Sb4 is shown to be biotinylated. (c) Development of an exo-cell assay. Utilization of the Sb4 substrate in conjunction with a small amount of CHAPSO detergent allows for real-time examination of γ-secretase activity directly from cells using ECL or homogenous time-resolved fluorescence (HTRF) detection methods in 96-well format.

Briefly, this recombinant substrate was based on a truncation of the APP substrate that encompassed the 76 C-terminal amino acids of APP. To this truncation, a maltose-binding protein (MBP) tag was cloned to assist with purification. Finally, an Avitag was also cloned onto the N-terminus of truncated APP (Fig. 1a). This avitag provides a mechanism whereby the recombinant protein can be directly biotinylated during over-expression in *Eschericia coli*. During induction of protein expression, biotin was added so that biotin ligase directly incorporated biotin into the recombinant γ-secretase substrate. This biotinylated recombinant substrate is referred to as Sb4 and was purified using an amylose resin column. After the recombinant protein was isolated, the sample was analyzed by LC-MS (Fig. 1b). The analysis showed that there were two species with molecular masses of 12,053 and 12,279, which correlated to non-biotinylated and biotinylated forms of substrate (calculated molecular masses were 12,050 and 12,276, respectively). Furthermore, it was determined that approximately 90% of purified Sb4 was biotinylated. We next attempted to apply the highly active Sb4 substrate to develop an assay capable of quantifying γ-secretase activity directly in cultured cells that would eliminate the need for stable transfection of substrate into cells or the isolation of membrane from large quantities of the cell line of interest.

**Figure 2:**
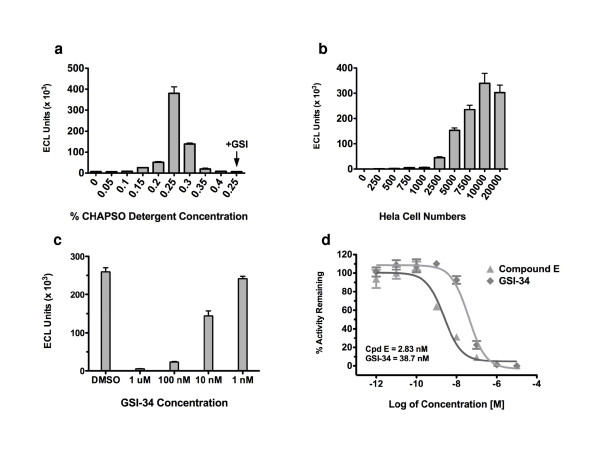
**Development of an exo-cell assay for quantification of γ-secretase activity in cells**. (a) Titration of CHAPSO detergent in the exo-cell assay. CHAPSO detergent was titrated to determine the optimal amount required for stimulating γ-secretase activity. The titration was performed using 10,000 HeLa cells and 1 μM Sb4 substrate. This reaction was incubated for 2.5 hours at 37°C. Supernatent was then collected and analyzed using ruthenylated G2-10* antibody. Activity was quantitated by measuring ECL. For each assay point n = 4, and s.d. is plotted. (b) Titration of the number of HeLa adenocarcinoma cells from which the exo-cell assay can detect γ-secretase activity. The indicated number of HeLa cells were seeded in a 96-well plate and allowed to attach overnight. The next day media was removed and replaced with fresh media containing 0.25% CHAPSO detergent, 1 μM Sb4 substrate, and DMSO or 1 μM Compound E to define background. Values plotted represent the activity quantified for each cell number assay point with GSI-defined background subtracted. For each assay point n = 4, and s.d. is plotted. (c) Dose-dependent inhibition of γ-secretase activity by GSI-34. HeLa cells were seeded 10,000 cells per well of 96-well plate. The cells were treated for 24 hours with the indicated concentration of GSI-34 inhibitor. Cells were then washed once with PBS and then the exo-cell assay was performed using 1 μM Sb4 substrate and 0.25% CHAPSO detergent. (d) IC_50 _values of distinct GSIs in extended exo-cell assay. IC_50 _values were obtained for 2 distinct GSI compounds using the extended exo-cell assay. For each data point n = 3, and s.d. is plotted.

In order to assay γ-secretase activity in cells, an exo-cell assay was designed that would allow for the evaluation of γ-secretase in real-time under diverse treatment conditions. Previously, Li et al. [10] had determined that in an *in vitro *γ-secretase assay, CHAPSO was superior to other detergents for promoting activity. Therefore, HeLa cells were first incubated with Sb4 substrate, as well as CHAPSO detergent as depicted in Fig. 1c. Specifically, HeLa cells were seeded in a 96-well plate and allowed to attach overnight. Then, media was removed, cells were washed once in PBS, and fresh media was added back containing 1 μM Sb4 substrate and CHAPSO detergent. The reaction mixture was incubated for 2.5 hours at 37°C. After cell debris was pelleted down by centrifugation, the resulting supernatant was used to examine γ-secretase-mediated product formation with G2-10 antibodies (Fig 1c). The amount of CHAPSO required for assaying activity in cells was first titrated, and it was determined that reproducible γ-secretase activity was detected within a range from 0.15% CHAPSO to an upper limit as high as 0.3% detergent. However, the greatest amount of activity was detected by using 0.25% CHAPSO (Fig. 2a), which is consistent with findings in a previously reported *in vitro *assay [10]. The activity at each of these concentrations could be attributed to γ-secretase in the HeLa cells as treatment with GSI abrogated cleavage of Sb4 (data only shown for 0.25% CHAPSO, Fig. 2a). Next, the sensitivity of the assay was evaluated by determining the lower limit of HeLa cell numbers needed to detect γ-secretase activity. The sensitivity of this assay was able to distinguish reproducible protease activity from as little as 2,500 HeLa adenocarcinoma cells with a signal to noise ratio greater than 5:1 (Fig. 2b). However, detectable activity was cell number dependent increasing from 1000 HeLa cells to 10,000 cells with the greatest activity found using 10,000 HeLa cells which produced a signal to noise ratio of approximately 125:1. The signal reaches its maximal amount at 10,000 HeLa cells and levels off at 20,000 HeLa cells due to the limiting substrate concentration in the assay.

### Evaluation of distinct γ-secretase inhibitors in the exo-cell assay

**Figure 3:**
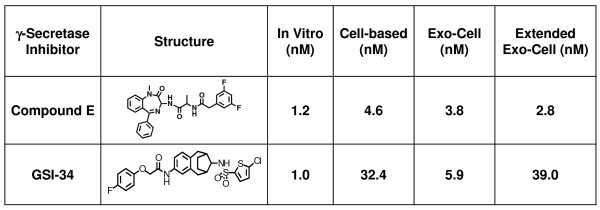
**Potency of γ-secretase inhibitors in various activity assays**. The potency of two structurally unique GSIs was assayed in four unique γ-secretase activity assays. IC_50 _values were determined from the dose response curves using a non-linear regression analysis in the Prism software. An *in vitro *assay was based on the one previously reported by Li et al. [10], except we utilized Sb4 substrate that eliminated the need for biotinylated antibody. The cell-based activity assay used N2A mouse neuroblastoma cells stably over-expressing APP and a biotinylated 4G8 antibody that binds the C-terminus of the β-amyloid peptide. The exo-cell assay incubated HeLa cells simultaneously with GSI, Sb4 substrate as well as 0.25% CHAPSO detergent prior to detecting substrate cleavage. Finally, the extended exo-cell assay first incubated HeLa cells with GSI for 24 hours. Subsequently, the cells were washed 1× in PBS and then incubated with Sb4 substrate and CHAPSO detergent. The assay was then carried out exactly as described for the original exo-cell method. All assays incorporate ruthenylated G2-10* antibody to detect 40-site cleavage of APP or recombinant Sb4 substrate and quantitated activity by measuring ECL.

Next, we examined the potency of various γ-secretase inhibitors in the exo-cell assay and compared the IC_50 _values to those from comparable *in vitro *and whole cell-based assays (Fig. 3). A range of inhibitors were assayed that included the benzodiazepine Compound E and a sulfonamide-based inhibitor referred to as GSI-34. These compounds all inhibit γ-secretase in the nanomolar range in our *in vitro *assay and their IC_50 _values are slightly elevated in the exo-cell assay (Fig. 3). It is worth noting that while the presented data employs ECL detection of cleaved substrate, we also successfully used homogeneous time-resolved fluorescence (HTRF) technology to detect cleaved substrate in our exo-cell assay (calculated IC_50 _values for Compound E and GSI-34 were 3.8 nM and 5.9 nM, respectively, using ECL. They were 5.4 nM and 5.4 nM using HTRF detection in the exo-cell assay).

### Detection of real-time γ-secretase inhibition

γ-Secretase inhibitors are currently being used as molecular probes and are being evaluated as potential therapeutics in Alzheimer's disease as well as for various neoplasms like T-cell acute lymphoblastic leukemia where γ-secretase-mediated Notch signaling is tumorigenic. We next modified the exo-cell assay so that our system can monitor real-time γ-secretase activity and inhibition. After HeLa cells were incubated with varying concentrations of GSI-34 or Compound E for 24 hours, media was removed and the cells were washed to remove excess unbound inhibitor. Fresh media containing only CHAPSO detergent and Sb4 substrate were placed back onto the cells and the exo-cell assay was then conducted as previously described. HeLa cells that were treated in this manner with GSI-34 show a dose-dependent inhibition of γ-secretase activity (Fig. 2c). This modified, extended treatment exo-cell assay is capable of quantifying remaining γ-secretase activity following drug treatment on virtually any cell type. The IC_50 _values for Compound E and GSI-34 were calculated using the extended exo-cell assay (Fig. 2d and Fig. 3). Comparing the potency of these unique GSIs in currently established *in vitro *and cell-based assays reveals that the extended exo-cell assay more closely mimics that witnessed in a cell-based γ-secretase assay that uses N2A mouse neuroblastoma cells stably expressing the APP substrate (N2A-APP) (Fig. 2d and Fig. 3). The IC_50 _values for Compound E and GSI-34 in the extended exo-cell assay were 2.83 nM and 38.7 nM respectively (Fig. 2d and Fig. 3) as compared to 4.6 nM and 32.4 nM, respectively, in the cell-based assay (Fig. 3) – both GSIs exhibited decreased potency in the cell-based and extended exo-cell assays as compared to their respective *in vitro *values. Regardless, the trend of decreasing potencies of GSIs in the extended exo-cell assay is similar to that witnessed in the stable N2A-APP cell-based system and this is likely due to the GSIs being incubated for 24 hours in the presence of a cellular environment that can affect compound half-life amongst other factors. These data show that our exo-cell assay can be used to evaluate the real-time status of γ-secretase activity in cell lines in a simple and sensitive manner.

**Figure 4:**
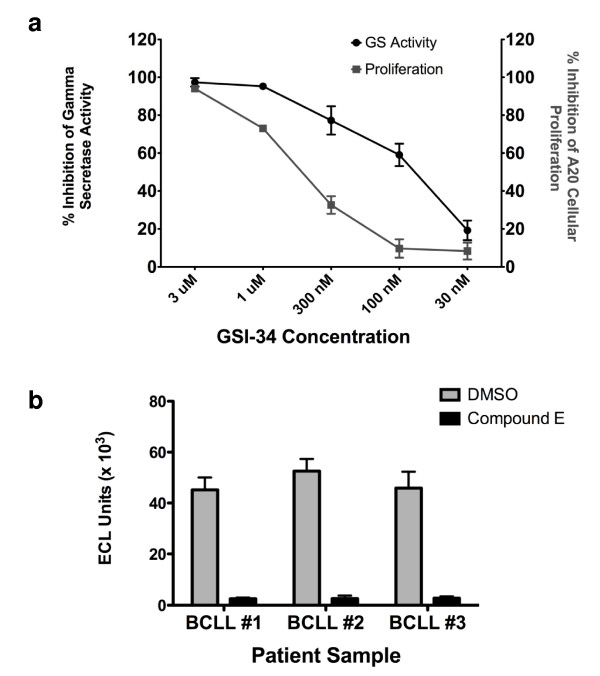
**Examination of real-time γ-secretase activity in A20 lymphoma and in primary B-CLL patient samples**. (a) Correlation between real-time inhibition of γ-secretase activity and GSI-mediated inhibition of A20 mouse lymphoma proliferation. Two 96-well plates were seeded with 50,000 A20 mouse lymphoma cells per well in 100 μl RPMI media. To each of these plates an additional 100 μl of media was added containing DMSO or GSI-34 to indicated final concentration. These plates were incubated for 48 hours at 37°C. Following this incubation, one plate was used in a real-time exo-cell assay to quantitate the real-time inhibition of γ-secretase in A20 cells. Briefly, A20 cells were pelleted and media removed. Fresh media containing 1 μM Sb4 substrate and 0.25% CHAPSO detergent were added and the exo-cell assay was performed. For each assay point n = 4, and s.d. is plotted. Additionally, to the other 96-well plate 2 μCi/ml [^3^H]thymidine was incubated with the cells for 5 hours. Following 5-hour incubation at 37°C, the amount of tritiated DNA was quantified on a β-counter. For each proliferation assay point n = 10, and s.d. is plotted. (b) Real-time γ-secretase activity in primary B-CLL patient samples. B-CLL cells were seeded in 96-well plate at a concentration of 50,000 cells per well. These were allowed to attach overnight. Subsequently, the media was removed and fresh media was added back that contained either DMSO or 1 μM Compound E inhibitor. This was incubated for 24 hours at 37°C. Cells were then washed once in PBS and exo-cell assay was performed as previously described. For each assay point n = 4, and s.d. is plotted.

Clearly, the extended exo-cell assay can be applied to quickly and efficiently quantitate the γ-secretase activity from any cultured cells in real-time. As such, we set out to utilize this novel assay to ascertain whether there exists a correlation between inhibition of γ-secretase and inhibition of cellular proliferation in a γ-secretase-dependent lymphoma line. Notch receptors require γ-secretase processing to release an intracellular fragment that translocates into the nucleus to transmit its signal. Multiple lymphoma lines have been shown to be dependent upon γ-secretase activity [14,15]. We have determined that the A20 mouse lymphoma line is sensitive to γ-secretase inhibition by GSI compounds (Fig. 4a) following 48 hours of treatment. Furthermore, we establish that there is a detectable correlation between this inhibition of cellular proliferation in A20 cells and inhibition of γ-secretase. Treatment of the A20 cell line with the three structurally unique, small molecule GSIs L-685,458, Compound E, and GSI-34 were all able to inhibit cellular proliferation (Fig. 4a for GSI-34; L-685,458 and Compound E data not shown) likely eliminating the possibility of an off-target, non-γ-secretase related effect. Interestingly, the data in Fig. 4a suggests that a small amount of remaining γ-secretase activity is sufficient to maintain cellular proliferation in this particular model system. For instance, 300 nM GSI-34 is able to inhibit approximately 80% of γ-secretase activity, yet this concentration only reduces cellular proliferation by 30% in the A20 mouse lymphoma model system. This data may help to explain the common finding that therapeutic levels of GSIs required to inhibit proliferation of Notch-dependent neoplastic cell lines are often far greater than *in vitro *IC_50 _values.

### Quantification of real-time γ-secretase activity in primary tumor samples

Lastly, it was not known whether this exo-cell method could be used to measure activity in primary samples from patients. Peripheral primary B-cell chronic lymphocytic leukemia cells (B-CLL) are arrested in the G_0 _phase of the cell cycle [16] and this condition makes it very difficult to assay γ-secretase activity in these primary cell samples. Additionally, it has been shown that Notch2 plays a role in the overexpression of CD23 in B-CLL and this may be related to the development of this neoplasm [17]. Therefore, the study of γ-secretase and Notch with regard to B-CLL biology has recently become an urgent issue. Stable transfection of substrate into a non-proliferating cell line is not a practical option and isolating enough B-CLL cells from a patient to prepare membrane fractions for use in *in vitro *assays is not feasible. However, the exo-cell assay now allows for the determination of protease activity quite easily. We have quantitated activity from three separate B-CLL patient samples and defined background activity for the assay in the presence of 1 μM Compound E (Fig. 4b) from 50,000 total B-CLL cells. Using the exo-cell assay we have been able to characterize γ-secretase activity in B-CLL patient samples for the first time. This previously would have been nearly impossible, but this assay makes it simple to detect protease activity in troublesome B-CLL patient samples in a few hours.

## Discussion

The application of our new exo-cell assay will have wide-ranging implications for the study of γ-secretase. While extensive efforts to characterize this protease have already been put forth due to its connection to Alzheimer's disease as well as numerous cancers, technical difficulties associated with the study of membrane enzymology has hindered much progress. The presented exo-cell assay will simplify the investigation of γ-secretase across different cell lines and tissue types as well as following diverse treatment conditions. Furthermore, this assay is able to efficiently evaluate the real-time status of γ-secretase from primary patient samples and completely eliminates the need for stable transfection of substrate or isolation of membrane. Not only will the quantification of protease activity in various cell lines now be simple, but this novel assay also provides a rapid means for evaluating the efficacy of potential therapeutics that affect γ-secretase in their target environment. For instance, γ-secretase inhibitors could previously be screened using *in vitro *assays against membrane isolated from the target cell line or tissue they were being used to treat, however, the real-time γ-secretase activity remaining in this system could not readily be evaluated following treatment. This is now an easy process that can be applied to nearly any cell line or tissue type and will require a nominal number of cells to do so. We are able to detect significant γ-secretase activity from as few as 2,500 HeLa adenocarcinoma cells using 0.25% CHAPSO detergent concentration. The detection of real-time activity is a significant development due to the central role of γ-secretase in numerous biological signaling pathways as well as in various disease states. Previously, it was impractical to evaluate γ-secretase activity in a system following cellular manipulation, however the exo-cell assay provides a simple means to assess the effect of GSI-application or other treatments on γ-secretase activity. Interestingly, we have shown that in a γ-secretase-dependent lymphoma line, cellular proliferation can be maintained by a minimal fraction of remaining γ-secretase activity. This finding may help to clarify why therapeutic concentrations of GSIs against lymphoma and other systems sensitive to inhibition of γ-secretase far exceed their calculated *in vitro *IC_50 _values. The real-time exo-cell assay simplifies the study of γ-secretase in diverse systems and will greatly facilitate the evaluation and development of novel therapies against Alzheimer's disease and cancers where γ-secretase plays a tumorigenic role as well as allow for the investigation of γ-secretase biology in systems where examination was not previously possible.

## Methods

### Production of γ-secretase recombinant substrate

Briefly, a DNA fragment encompassing amino acids 620–695 of the 695-aa isoform of APP as well as a maltose binding protein tag was cloned into the pIAD16 prokaryotic vector [18]. Additionally, there was an Avitag also incorporated into this vector. Avitag, a specific 15-residue peptide, is recognized by biotin ligase that specifically catalyzes an attachment of biotin to the lysine residue within the Avitag. The protein was then co-expressed in *Eschericia coli *with the pACYC184 biotin ligase plasmid. IPTG at 0.1 mM was used to induce expression of Sb4 as well as biotin ligase at 20°C for 5 hours in the presence of 50 μM biotin. Biotin ligase directly biotinylates the avitag during protein expression. Sb4 was ultimately purified using an amylose resin column, eluted with excess maltose and thrombin-cleaved to remove maltose-binding protein from the purified substrate. The biotinylation of recombinant substrate was confirmed by LC-MS.

### Real-time exo-cell γ-secretase activity assay

Cells were seeded at their indicated concentration in 96-well plates and allowed to attach overnight (Fig. 1c). The next day, media was removed and cells were washed once with PBS. Fresh media was then added containing 0.25% CHAPSO detergent, Sb4 substrate to a final concentration of 1 μM, and 1% DMSO or γ-secretase inhibitor. This was incubated for 2.5 hours at 37°C. Media was removed and cell debris was pelleted from this media for 5 min. at 3,500 rpm's. Supernatant was then added to ruthenylated G2-10* antibody that recognizes cleaved product, but not uncleaved substrate. This was incubated for an additional 2 hours at room temperature. Finally, magnetic streptavidin beads were added to a final concentration of 80 μg/ml and incubated for 30 min. at room temperature. Assay buffer was added to the samples and γ-secretase-mediated cleavage of substrate was monitored using electrochemiluminescence (ECL) [10]. Protocol for HTRF detection can be found below.

Conversely, an extended exo-cell assay was performed whereby cells were seeded and allowed to attach overnight as previously described. However, the next day media was removed and replaced with fresh media containing 1% DMSO or γ-secretase inhibitor. These were incubated for 24 hours at 37°C. Following this incubation, the cells were washed once with PBS and fresh media containing detergent and substrate were added. From this point on, the exo-cell assay as described above was followed.

Homogeneous time-resolved fluorescence (HTRF) was also incorporated for the exo-cell assay detection of cleaved substrate. The exo-cell assay proceeded as previously described except that media supernatent was collected and 10 μl of supernatent was added to 10 μl of HTRF detection mix. The HTRF detection mix contained 1 nM G2-10 antibody, 2 nM anti-mouse IgG cryptate entity and 15 nM Streptavidin-XL665 flourophore. These were all dissolved in HTRF detection buffer which was 50 mM phosphate buffer, pH 7.0, 0.2% BSA and 0.8 M potassium fluoride. Exo-cell supernatent and HTRF detection mix were incubated together for 5 hours in a 384-well plate and then activity was measured by stimulating at 337 nm and reading at 665 nm.

### In vitro and cell-based γ-secretase assays

The *in vitro *and cell-based γ-secretase assays were performed similarly as previously described [10]. Briefly, the recombinant Sb4 substrate was incubated for 2.5 hours at 37°C in pH 7.0 PIPES buffer in the presence of 0.25% CHAPSO detergent and solubilized γ-secretase at a final concentration of 40 ng/ul. The detection of cleaved substrate was determined using ruthenylated G2-10* antibody. Due to biotinylation of the Sb4 substrate, this eliminated the additional use of 6E10 biotinylated antibody from the original protocol.

The cell-based assay utilized N2A mouse neuroblastoma cells that stably overexpress the amyloid precursor protein. These were incubated with γ-secretase inhibitors in a final concentration of 1% DMSO for 24 hours. Following incubation, the supernatant was removed from the cells and assayed for Aβ 40-site cleaved APP product using ruthenylated G2-10* antibody as well as biotinylated 4G8 antibody.

### Lymphoma proliferation assay

A20 mouse lymphoma cells were seeded in a 96-well plate at a concentration of 5 × 10^5 ^cells/ml in 100 μl RPMI media containing 2% fetal bovine serum. An additional 100 μl of media containing DMSO or γ-secretase inhibitor was added and incubated for 48 hours at 37°C. After this incubation, the cells were incubated for 5 hours with 2 μCi/ml [^3^H]thymidine at 37°C. Proliferative response was then evaluated by harvesting the tritiated DNA from cells using a Skatron cell harvester and proliferation assessed as a function of [^3^H]thymidine incorporation measured on a β-counter.

### Primary B-CLL patient sample isolation

Primary samples were obtained from patients diagnosed with B-cell chronic lymphocytic leukemia who were untreated. Written informed consent was obtained from each patient in accordance with the guidelines of the Institutional Review Board of Memorial Sloan Kettering Cancer Center and the Declaration of Helsinki. Peripheral blood mononuclear cells (PBMCs) were isolated using standard Ficoll-Hypaque density gradient and subsequently stored in liquid nitrogen. Prior to use in assay, samples were thawed and resuspended in RPMI media and allowed to attach overnight at 37°C. The extended exo-cell assay was then performed as described above.

## Competing interests

The authors declare that they have no competing interests.

## Authors' contributions

CS. and YML initiated the research project, designed the experiments, and prepared the manuscript. CS carried out all biochemical and cell-based experiments. YT designed and produced the Sb4 substrate. MF obtained B-CLL patient samples as well as offered critical analysis of the scientific design and manuscript.
